# Interfacial Design of Ti_3_C_2_T_x_ MXene/Graphene Heterostructures Boosted Ru Nanoclusters with High Activity Toward Hydrogen Evolution Reaction

**DOI:** 10.1002/advs.202310013

**Published:** 2024-03-29

**Authors:** Xu Yu, Yong Li, Chengang Pei, Yanhui Lu, Jung Kyu Kim, Ho Seok Park, Huan Pang

**Affiliations:** ^1^ School of Chemistry and Chemical Engineering Yangzhou University Yangzhou 225002 P. R. China; ^2^ Department of Chemical Engineering College of Engineering Sungkyunkwan University 2066, Seobu‐ro, Jangan‐gu Suwon‐si Gyeonggi‐do 16419 Republic of Korea

**Keywords:** graphene, hierarchical structure, hydrogen evolution reaction, MXene, ruthenium

## Abstract

The development of a cost‐competitive and efficient electrocatalyst is both attractive and challenging for hydrogen production by hydrogen evolution reaction (HER). Herein, a facile glycol reduction method to construct Ru nanoclusters coupled with hierarchical exfoliated‐MXene/reduced graphene oxide architectures (Ru‐E‐MXene/rGA) is reported. The hierarchical structure, formed by the self‐assembly of graphene oxides, can effectively prohibit the self‐stacking of MXene nanosheets. Meanwhile, the formation of the MXene/rGA interface can strongly trap the Ru^3+^ ions, resulting in the uniform distribution of Ru nanoclusters within Ru‐E‐MXene/rGA. The boosted catalytic activity and underlying catalytic mechanism during the HER process are proved by density functional theory. Ru‐E‐MXene/rGA exhibits overpotentials of 42 and 62 mV at 10 mA cm^−2^ in alkaline and acidic electrolytes, respectively. The small Tafel slope and charge transfer resistance (*R*_ct_) values elucidate its fast dynamic behavior. The cyclic voltammetry (CV) curves and chronoamperometry test confirm the high stability of Ru‐E‐MXene/rGA. These results demonstrate that coupling Ru nanoclusters with the MXene/rGA heterostructure represents an efficient strategy for constructing MXene‐based catalysts with enhanced HER activity.

## Graphical abstract

Ru‐E‐MXene/rGA, constructed by immobilizing Ru nanoclusters on an exfoliated‐MXene/graphene interface, shows exceptional catalytic HER activity, attributing to the controllable morphological structure and the rational interfacial design. The hierarchical structure can expose abundant active sites, provide pathways for electrolyte ion diffusion, and facilitate charge transfer. Meanwhile, the formed heterostructure with strong chemical interaction at the interfaces can boost the catalytic active sites to improve the electrocatalytic HER performance.

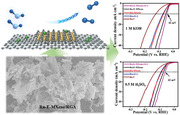

## 1 Introduction

Hydrogen energy is increasingly regarded as a promising clean energy carrier with the potential to replace traditional fossil fuels, owing to its high energy density, pollution‐free combustion products, and abundant reserves.^[^ ^1^ ^]^ Many efficient efforts to produce hydrogen energy have been widely studied, and electrocatalytic water splitting is considered a promising method due to its carbon emission‐free nature, production of high‐purity hydrogen, and scalability.^[^ ^2^ ^]^ The intrinsic properties of the electrocatalyst play an important role in the electrolysis of water to produce hydrogen. Therefore, it is of great importance to develop an efficient and stable electrocatalyst for hydrogen evolution reaction.^[^ ^3^ ^]^

Recently, platinum (Pt) catalysts are considered as the most active catalysts at all pH values for hydrogen evolution reaction (HER). However, their high price, high scarcity, and low abundance of resources have hindered large‐scale utilization.^[^ ^4^ ^]^ Ruthenium (Ru), as a good substitute of Pt, owns a similar hydrogen bond energy and low barrier of water decomposition,^[^ ^5^ ^]^ but its high binding energy can lead to the aggregation of Ru nanoclusters to decrease the HER activity.^[^ ^6^ ^]^ To solve this emergent issue, the introduction of highly conductive material as substrate is a promising strategy to guarantee the uniform distribution of Ru and reduce the cost of catalysts with excellent HER activity.^[^ ^2^, ^7^ ^]^ Many efforts on carbon‐based nanomaterials as efficient support, such as graphene, carbon nanotubes (CNTs), carbon nitride, and carbon quantum dots, have been demonstrated to inhibit the aggregation of Ru nanoclusters with maintaining excellent catalytic activity.^[^ ^8^ ^]^ Therefore, the development of low‐cost Ru‐based catalysts with excellent electrical conductivity and high catalytic activity has been pursued to satisfy the practical application.^[^ ^9^ ^]^

MXene, as a layered 2D transition metal carbides/ nitrides, has been proven as an attractive electrocatalyst for HER, due to their abundant surface termination groups (‐O, ‐OH, or ‐F) and high hydrophilicity.^[^ ^10^ ^]^ The mono‐ or few‐layered MXene offers increased exposure of surface active sites, while the hybridization of MXene with noble metal leads to improvement in the electrocatalytic HER performance,^[^ ^10^, ^11^ ^]^ such as Ru@1T‐MoS_2_‐MXene catalyst^[^ ^12^ ^]^ and Ru/Mo_2_CT_x_ catalyst.^[^ ^13^ ^]^ The negatively charged surface of MXene can strongly hold positively charged metal ions, which can effectively hinder the agglomeration of metal nanoparticles, reduce the resistance between the active sites and the carriers, promote electron transfer and enhance HER activity.^[^ ^5^, ^11^, ^14^ ^]^ However, the self‐restacking issue and poor stability of MXene can hamper the exposure of the catalytic active sites due to their high surface energy.^[^ ^15^ ^]^ Recently, the construction of catalyst with the heterostructure has been demonstrated to show the improved catalytic activity by adjusting the electron transfer at the interface.^[^ ^16^ ^]^ MXene, as the second catalytic phase, is introduced to the Ru/carbon system to controllable design the 2D/2D heterostructure. The rational design of interface structure not only guarantees the distribution of MXene on the catalyst surface but also improves the electrocatalytic performance with low Ru content.^[^ ^15^, ^17^ ^]^ Therefore, the construction of the controllably morphological structure of HER electrocatalyst in full pH range becomes emergent and important to satisfy the practical application.

Herein, the Ru nanoclusters coupled hierarchical exfoliated‐MXene/reduced graphene oxides architectures (Ru‐E‐MXene/rGA) were prepared via a one‐step solvothermal method under the reductive properties of ethylene glycol. The hierarchical structure is constructed by the self‐assembly of graphene nanosheets via π–π configuration. Meanwhile, the deposition of MXene and Ru on graphene can significantly prevent the self‐stacking of 2D MXene nanosheets and the aggregation of Ru nanoclusters. Due to the controllable morphology and adjusted surface chemistry, Ru‐E‐MXene/rGA exhibits excellent HER performance in a wide pH range, such as the low overpotential of 42 and 62 mV in 1 M KOH and 0.5 M H_2_SO_4_ at 10 mA cm^−2^, as well as the low Tafel slope and small charge transfer resistance.

## 2 Results and Discussion

**Figure 1:**
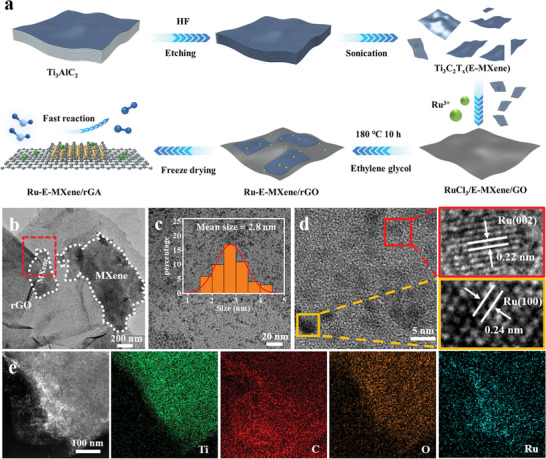
a) Schematic illustration of the synthetic process of Ru‐E‐MXene/rGA. b) TEM image and c,d) HR‐TEM images of Ru‐E‐MXene/rGA. The inset in c) represents the size distribution of Ru nanoclusters. e) STEM and the related elemental mapping images of Ru‐E‐MXene/rGA.

The synthetic process of Ru nanoclusters coupled with hierarchical exfoliated‐MXene/reduced graphene oxides architectures (Ru‐E‐MXene/rGA) is illustrated in **Figure** **1**a. In brief, the bulk MAX was etched by removing the Al layer to obtain the accordion‐like structured MXene, as confirmed by scanning electron microscopy (SEM) in Figure S1a and S1b (Supporting Information), transmission electron microscopy (TEM) in Figure S2 (Supporting Information) and X‐ray diffraction (XRD) in Figure S3 (Supporting Information). The exfoliation of layered MXene is essential to obtain the high‐quality mono‐ or few layered MXene nanosheets following the further freeze‐drying treatment (Figure S1c, Supporting Information). The exfoliated MXene (E‐MXene) powder and graphene oxides were dispersed into ethylene glycol, and the negatively charged dispersion was determined by the abundant functional groups on the surface of MXene/GO. The quality of the RuCl_3_/MXene/GO mixture is important to guarantee the uniform distribution of small‐sized Ru nanoclusters and prevent the re‐stacking of MXene nanosheets. During the hydrothermal process, GO and Ru^3+^ are in‐situ reduced by the ethylene glycol, and Ru‐E‐MXene/rGA with hierarchical structure is finally obtained after the freeze‐drying process (Figure S1d, Supporting Information). As confirmed by nitrogen adsorption/desorption isotherm in Figure S4 and Table S1 (Supporting Information) the specific surface area of Ru‐E‐MXene/rGA is 41.3 m^2^ g^−1^ with the average pore size of 3.7 nm and pore volume of 0.15 cm^3^ g^−1^.

The morphological structure of Ru‐E‐MXene/rGA was further characterized by TEM. During the exfoliation process, the accordion‐like MXene was stripped into a mono‐ or few‐layered structure, and the size of MXene nanosheets decreased, facilitating their immobilization onto the graphene surface (Figure 1b). Due to the negatively charged surface of MXene and graphene, the positively charged Ru^3+^ can be strongly trapped and in situ reduced during the hydrothermal process.^[^ ^18^ ^]^ The generation of E‐MXene/rGA heterostructures is beneficial for enhancing the electrical conductivity and preventing the aggregation of Ru nanoclusters, thus facilitating the electrocatalytic process.^[^ ^19^ ^]^ Figure 1c shows the uniform distribution of Ru nanoclusters on the surface of MXene and graphene. The average size is ≈2.8 nm for Ru‐E‐MXene/rGA (inset of Figure 1c), which is much smaller than that of Ru‐MXene and Ru‐rGA (Figure S5, Supporting Information). The small‐sized Ru nanoclusters can expose more accessible active sites and facilitate charge transfer at the MXene/graphene interfaces, which is a great benefit to the improvement of catalytic performance.^[^ ^16a^ ^]^ As shown by the high‐resolution TEM images in Figure 1d, the two‐finger lattice spacings of 0.22 and 0.24 nm correspond to the (002) and (100) planes of crystal Ru nanoclusters,^[^ ^20^ ^]^ implying the successful reduction of Ru^3+^ ions, which is consistent with the selected area electron diffraction (SAED) in Figure S6 (Supporting Information). The elemental mapping confirms the uniform distribution of Ru, Ti, C, and O elements for Ru‐E‐MXene/rGA in Figure 1e, which agrees with the energy dispersive spectroscopy (EDS) result in Figure S7 (Supporting Information). A clear boundary between C, O, and Ti elements implies the immobilization of MXene on graphene, and their outline is well‐matched with the STEM image. These results demonstrate that the small‐size Ru nanoclusters are successfully anchored on MXene/rGA heterostructures.

**Figure 2:**
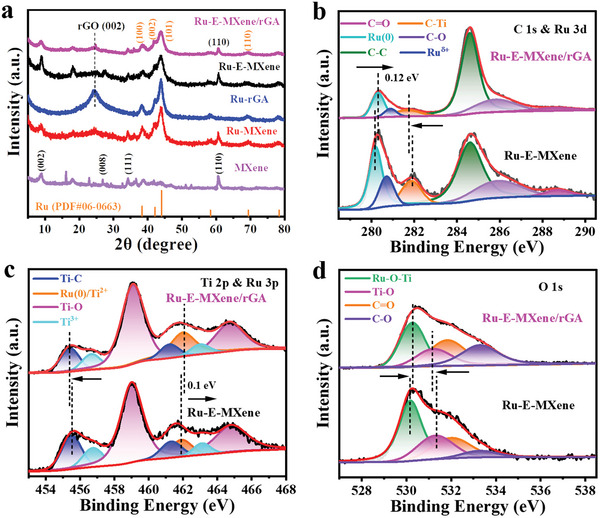
a) XRD patterns of Ru‐E‐MXene/rGA, Ru‐E‐MXene, Ru‐MXene, and Ru‐rGA. The high‐resolution XPS spectra of Ru‐E‐MXene/rGA and Ru‐E‐MXene, b) C 1s & Ru 3d, c) Ti 2p & Ru 3p and d) O1s for Ru‐E‐MXene/rGA and Ru‐E‐MXene.

The crystallinity of the Ru‐E‐MXene/rGA was analyzed by XRD in **Figure** **2**a. Ru‐E‐MXene/rGA exhibits the diffraction peaks at 38.4°, 41.8°, and 44.0° can be indexed to the (100), (002), and (101) planes of Ru metal (PDF #06‐0663), respectively, indicating the reduction of Ru^3+^ to Ru nanoclusters. Especially, Ru‐E‐MXene/rGA owns a weaker peak intensity and a larger Full‐Width Half Maxima (FWHM) of diffraction peaks of Ru crystal than those of Ru‐E‐MXene, Ru‐MXene, and Ru‐rGA, implying a smaller size of Ru nanoclusters.^[^ ^21^ ^]^ A broad diffraction peak for Ru‐E‐MXene/rGA can be observed at 24.3° corresponding to the (002) plane of reduced graphene, which is much weaker than that of Ru‐rGA.^[^ ^22^ ^]^

To analyze the electronic structure and chemical states of the Ru‐E‐MXene/rGA, X‐ray photoelectron spectroscopy (XPS) was performed. The binding energy of all peaks was calibrated based on the C 1s peak at 284.6 eV. The survey scan confirms that Ru‐E‐MXene/rGA is composed of C, Ti, O, Ru, and F elements, and the existence of F element is from the etching process of MXene (Figure S8 and Table S2, Supporting Information). For the C 1s & Ru 3d XPS spectra of Ru‐E‐MXene/rGA in Figure 2b, four primary peaks can be deconvoluted at 281.6, 284.6, 285.8, and 288.6 eV corresponding to Ti─C, C−C, C−O, and C═O bonds, respectively.^[^ ^7a^ ^]^ In addition, one peak at 280.3 eV is attributed to Ru (0) implying the formation of Ru nanoclusters, and one peak at 280.8 eV is indexed to Ru^&+^ species owing to the partial oxidation of catalyst in the air.^[^ ^8^, ^23^ ^]^ In comparison with Ru‐E‐MXene, the positive shift of Ru (0) and negative shift of Ti‐C result from the strong electronic interaction and electron transfer at Ru‐Ti_3_C_2_T_x_/rGA interface.^[^ ^24^ ^]^ Meanwhile, the Ru (0) peak is positive shift in contrast to Ru‐rGA in Figure S9 (Supporting Information) attributing to the formation of MXene/rGA heterostructure. For Ti & Ru spectra in Figure 2c, three pair of peaks at 455.3/461.2, 456.6/463, and 459.1/464.7 eV are assigned to the Ti‐C, Ti^3+^, and Ti‐O for Ru‐E‐MXene/rGA, respectively.^[^ ^25^ ^]^ And a peak at 461.4 eV is attributed to Ti^2+^ or Ru (0) because of their similar energy range with each other.^[^ ^26^ ^]^ The Ti‐C bond is negative shift, and the Ru (0) bond is positive shift implying the electron transfer from Ru to MXene. The Ti─C bond from C1s and Ti 2p spectra proves the existence of Ti_3_C_2_T_x_ and the formation of MXene/rGA interfaces. The fitted O 1s spectra of Ru‐E‐MXene/rGA (Figure 2d) show four peaks at 530.3, 531.2, 531.8, and 533.3 eV, which can be assigned to Ru‐O‐Ti, Ti‐O, C = O, and C‐O, respectively.^[^ ^11^, ^27^ ^]^ It can be found that a slight shift of Ru‐O‐Ti and Ti‐O peaks is consistent with the change trend of Ti 2p and Ru 3d. The XRD and XPS results fully demonstrate the successful synthesis of the Ru‐E‐MXene/rGA. Noticeably, the shift of the Ru (0) peak for Ru‐E‐MXene/rGA suggests the existence of electron transfer at the interfaces and the strong electrostatic interaction between Ru and conductive MXene/rGA matrix, which can weaken the H* adsorption energy and improve the electrocatalytic performance.^[^ ^12^, ^28^ ^]^

**Figure 3:**
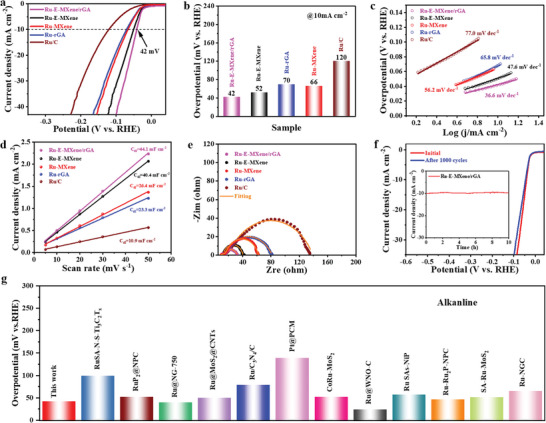
Electrocatalytic HER performance in 1 M KOH. a) Polarization curves and b) overpotentials of Ru‐E‐MXene/rGA, Ru‐E‐MXene, Ru‐MXene, Ru‐rGA and Ru/C at 10 mA cm^−2^. c) Tafel slopes, d) *C*_dl_ values and e) Nyquist plots of Ru‐E‐MXene/rGA, Ru‐E‐MXene, Ru‐MXene, Ru‐rGA and Ru/C. f) Comparison of the polarization curve of Ru‐E‐MXene/rGA before and after 1000 cycles (inset: chronoamperometry test). g) Performance comparison of Ru‐E‐MXene/rGA and recently reported HER electrocatalysts in 1 M KOH, concerning in Table S6 (Supporting Information).

The electrochemical HER performances of all catalysts were studied by a three‐electrode system in 1.0 M KOH. **Figure** **3**a shows the linear sweep voltammetry (LSV) curves of Ru‐E‐MXene/rGA, Ru‐E‐MXene, Ru‐MXene, Ru‐rGA and Ru/C in a N_2_‐saturated KOH solution at 5 mV s^−1^. An overpotential of 42 mV is required for Ru‐E‐MXene/rGA to afford the current density of 10 mA cm^−2^ (Figure 3b), which is lower than that of Ru‐E‐MXene (52 mV), Ru‐MXene (66 mV), Ru‐rGA (70 mV) and Ru/C (120 mV), respectively, Tafel slope, as an important factor, was applied to study the kinetic behavior during HER process, and a smaller value of Tafel slope means a faster kinetic behavior. In Figure 3c, Ru‐E‐MXene/rGA owns a smaller value of Tafel slope (36.6 mV dec^−1^) than that of Ru‐E‐MXene (47.6 mV dec^−1^), Ru‐MXene (56.2 mV dec^−1^), Ru‐rGA (65.8 mV dec^−1^) and Ru/C (77.0 mV dec^−1^), implying a higher catalytic HER activity and faster HER reaction kinetics for Ru‐E‐MXene/rGA. Meanwhile, Ru‐E‐MXene/rGA shows a typical Volmer–Tafel reaction mechanism, including the Tafel reaction as a rate‐determining step.^[^ ^29^ ^]^ To investigate the active surface area of Ru‐E‐MXene/rGA, the electrochemical surface area (ECSA) was determined by the double‐layer capacitances (*C*_dl_), which could be calculated by CV curves in the non‐faradic region (Figure S10 and Table S3, Supporting Information). At the scan rate from 5 to 50 mV s^−1^, the *C*_dl_ values are calculated to 44.1, 40.4, 26.4, 23.3, and 10.9 mF cm^−2^ for Ru‐E‐MXene/rGA, Ru‐E‐MXene, Ru‐rGA, Ru‐MXene and Ru/C in Figure 3d. A large *C*_dl_ value for Ru‐E‐MXene/rGA suggests more exposed active sites.

Furthermore, the electrochemical impedance spectroscopy (EIS) was carried out to verify the kinetic behavior of Ru‐E‐MXene/rGA at the electrode/electrolyte surface during the HER process in Figure 3e. The diameter of the semicircle in the mid‐frequency region of the Nyquist plot represents the charge transfer resistance (*R*_ct_), which can reflect the charge transfer kinetics.^[^ ^30^ ^]^ By fitting the Nyquist plots by an electrical equivalent circuit (Figure S11 and Table S4, Supporting Information), Ru‐E‐MXene/rGA owns a smaller *R*_ct_ value (18.4 Ω) than those of the control samples implying its faster electron transfer and a superior kinetic behavior during HER process, which is attributed to the increased electrical conductivity by introducing reduced graphene and the chemical interaction at MXene/rGA interfaces. The long‐term stability of Ru‐E‐MXene/rGA was measured by the CV curves for 1000 cycles and chronoamperometry (CA) test for 10 h. By comparing the CV curves recorded before and after 1000 CV cycles in Figure 3f, a negligible shift of curve can be observed and the loss of overpotential is only 4 mV, demonstrating a good catalytic stability of Ru‐E‐MXene/rGA. As further confirmed by CA test for 10 h (inset of Figure 3f; Figure S12, Supporting Information), only 80% maintenance of current density for Ru‐E‐MXene and no obvious degradation of current density for Ru‐E‐MXene/rGA imply excellent durability of Ru‐E‐MXene/rGA and the minimal Ru content can be found in the electrolyte after CA test (Table S5, Supporting Information). The excellent electrocatalytic HER performance of Ru‐E‐MXene/rGA is superior to other reported Ru‐based catalysts in Figure 3g and Table S6 (Supporting Information) attributing to the synergistic effect of hierarchical structure and the small‐sized Ru nanoclusters anchoring on the MXene/rGA interfaces.

**Figure 4:**
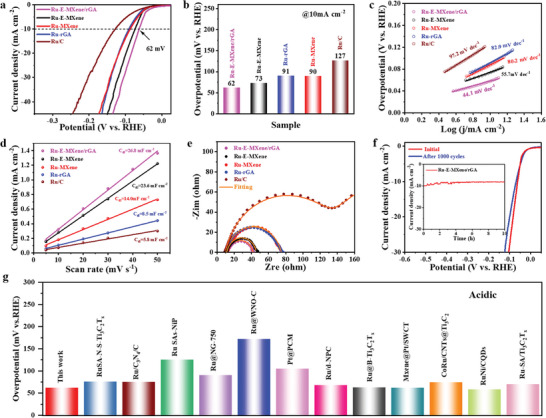
Electrocatalytic HER performance tests in 0.5 M H_2_SO_4_ media a) Polarization curves and b) overpotentials of Ru‐E‐MXene/rGA, Ru‐E‐MXene, Ru‐MXene, Ru‐rGA and Ru/C at 10 mA cm^−2^. c) Tafel slopes, d) *C*_dl_ values and e) Nyquist plots of Ru‐E‐MXene/rGA, Ru‐E‐MXene, Ru‐MXene, Ru‐rGA and Ru/C. f) Comparison of the polarization curve of Ru‐E‐MXene/rGA before and after 1000 cycles (inset: chronoamperometry tests). g) Performance comparison of Ru‐E‐MXene/rGA and recently reported HER electrocatalysts in 0.5 M H_2_SO_4_, concerning Table S9 (Supporting Information).

To further prove the excellent superior electrocatalytic HER performance, Ru‐E‐MXene/rGA was also probed by a three‐electrode configuration in 0.5 M H_2_SO_4_. **Figure** **4**a shows the LSV curves of all catalysts, and the overpotential of Ru‐E‐MXene/rGA at 10 mA cm^−2^ (62 mV) is smaller than that of Ru‐E‐MXene (73 mV), Ru‐MXene (90 mV), Ru‐rGA (91 mV), and Ru/C (127 mV) in Figure 4b. As shown in Figure 4c, Ru‐E‐MXene/rGA shows a smaller Tafel slope of 44.1 mV dec^−1^ than that of the Ru‐E‐MXene (55.7 mV dec^−1^), Ru‐MXene (80.2 mV dec^−1^), Ru‐rGA (82.9 mV dec^−1^) and Ru/C (97.2 mV dec^−1^), indicating a favorable reaction kinetics and a Volmer‐Heyrovsky reaction mechanism for Ru‐E‐MXene/rGA during the HER process in acidic electrolyte.^[^ ^31^ ^]^ The HER process in acidic electrolyte is predominated by the intermediate hydrogen adsorption kinetics without the water dissociation process, which can be accelerated by Ru in alkaline media.^[^ ^5c^ ^]^ The *C*_dl_ values calculated by CV curve in non‐faradic region in Figure S13 (Supporting Information) and the ECSA value and are listed in Table S7 (Supporting Information). In Figure 4d, Ru‐E‐MXene/rGA presents a larger *C*_dl_ value (26.8 mF cm^−2^) than that of Ru‐E‐MXene, Ru‐MXene, Ru‐rGA, and Ru/C, revealing that the Ru‐E‐MXene/rGA owns abundant electrocatalytic active sites. Moreover, Ru‐E‐MXene/rGA shows the smallest *R*_ct_ value among all the catalysts revealing its outstanding charge transfer ability during the HER process (Figure 4e and Table S8, Supporting Information). The electrochemical stability of the Ru‐E‐MXene/rGA in the acidic electrolyte was evaluated by CV for 1000 cycles and CA for 10 h. After 1000 CV cycles, the polarization curve for Ru‐E‐MXene/rGA only shows a negligible negative shift (Figure 4f) and no obvious decrease of the specific current density after the CA test for 10 h (inset of Figure 4f), elucidating that Ru‐E‐MXene/rGA possesses excellent catalytic stability in acidic electrolyte and the minimal Ru content can be found in the electrolyte after CA test (Table S5, Supporting Information). The excellent HER performance of Ru‐E‐MXene/rGA in acidic electrolyte is compared with the reported lectures in Figure 4g and Table S9 (Supporting Information). Meanwhile, the overpotentials of Ru‐E‐MXene/rGA in both alkaline and acidic electrolyte are smaller than these of Ru/C catalyst at a high current density of 200 mA cm^−2^ in Figure S14 (Supporting Information). In comparison to its initial state, the current density Ru‐E‐MXene/rGA remain ≈94.1% in alkaline electrolyte after CA test for 48 h in Figure S15 (Supporting Information). The Faraday efficiency of Ru‐E‐MXene/rGA in both acidic and alkaline electrolytes was evaluated by comparing the experimental amount of hydrogen production with the theoretical amount of hydrogen gas in Figure S16 (Supporting Information). The Faradic efficiency is close to 100% in both acidic and alkaline electrolytes, determined by the well‐matched experimental and theoretical values.

The excellent HER performance of Ru‐E‐MXene/rGA in both acidic and alkaline electrolytes can be assigned to the synergistic effect of morphological structure and modified surface chemistry. 1) The hierarchical structure can provide a large surface area, abundant active sites, and lots of pathways for fast ion diffusion; 2) the uniform distribution of small‐sized Ru nanoclusters and the inhibition of re‐stacking of layered MXene can increase active sites of catalyst; 3) the interaction at MXene/rGA interface can strong trap Ru nanoclusters and increase the conductivity of catalyst to enhance the electrocatalytic performance.

**Figure 5:**
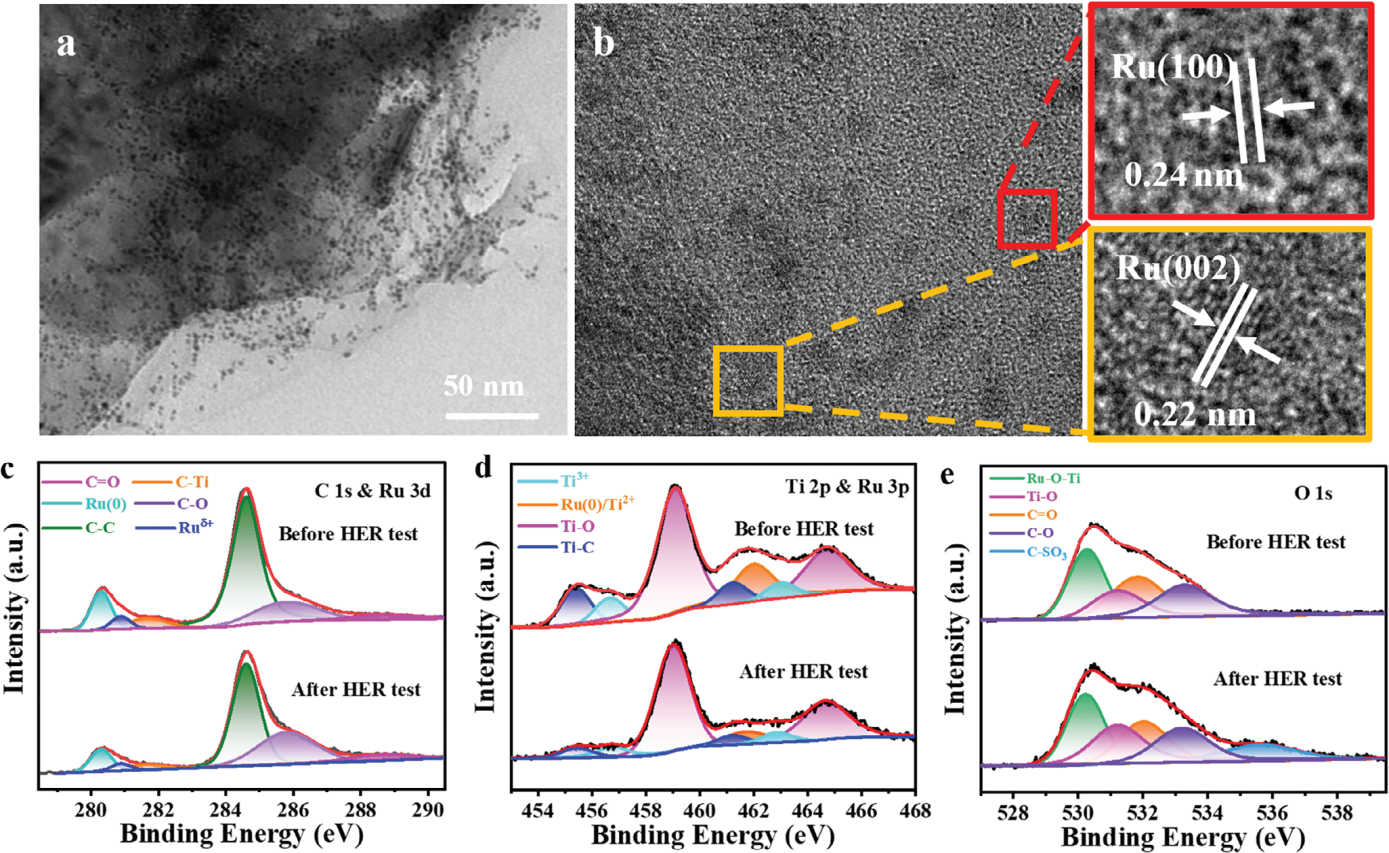
a) TEM image of Ru‐E‐MXene/rGA after stability test in 1 M KOH. b) HR‐TEM images of Ru‐E‐MXene/rGA after stability test in 1 M KOH. XPS spectra of c) C 1s & Ru 3d, d) Ti 2p & Ru 3p and e) O 1s for Ru‐E‐MXene/rGA before and after stability test in 1 M KOH.

The change of morphological and surface chemistry for Ru‐E‐MXene/rGA after the CA test in alkaline electrolyte was compared with its pristine state. As the TEM image is shown in **Figure** **5**a, no aggregation of Ru nanoclusters can be found and the obscure surface of Ru‐E‐MXene/rGA can be assigned to the surface oxidation during the HER process. The change of surface chemistry is probed by XPS. For the full scan of XPS spectra, a strong peak intensity of F element after the CA test is attributed to the residue of Nafion as the binder (Figure S17, Supporting Information). For C 1s and Ru 3d spectra in Figure 5c, the deconvoluted Ru (0) and Ti‐C peaks of Ru‐E‐MXene/rGA after the CA test become weaker than the pristine state, and the peak intensity of C─O bond becomes stronger, resulting from the partial oxidation of catalyst. As further confirmed by Ti 2p and Ru 3p spectra in Figure 5d, the weak strength of Ti─C and Ru (0) bonds after CA test and the strong strength of Ti─O bond after CA test are attributed to the formation of Ti‐based oxides on the surface of MXene during the HER process.^[^ ^32^ ^]^ In particular, the surface can be converted into oxide or hydroxide species in the first stage and become steady afterward during the long‐term HER process.^[^ ^33^ ^]^ The increased peak intensity of metal─O bond after the CA test was verified by O 1s spectra in Figure 5e and the peak of C─SO_3_ bond comes from the Nafion employed for the catalyst ink fabrication

To further determine the high HER activity of Ru‐E‐MXene/rGA in alkaline and acidic solutions, density functional theory calculations were performed. The HER activity is strongly correlated with the Gibbs free energy (ΔG_H*_) of hydrogen adsorption on the surface of the catalyst and the value of ΔG_H*_ implies the optimization of hydrogen atom adsorption/desorption. The ΔG_H*_ is close to zero implying a good electrocatalytic HER activity.^[^ ^34^ ^]^ However, the low ΔG_H*_ value leads to a slow hydrogen release step due to the strong chemical bond with the hydrogen atom and the high ΔG_H*_ value causes an excessive H* adsorption during the electron transfer step, which is unfavorable for the HER reaction.

**Figure 6:**
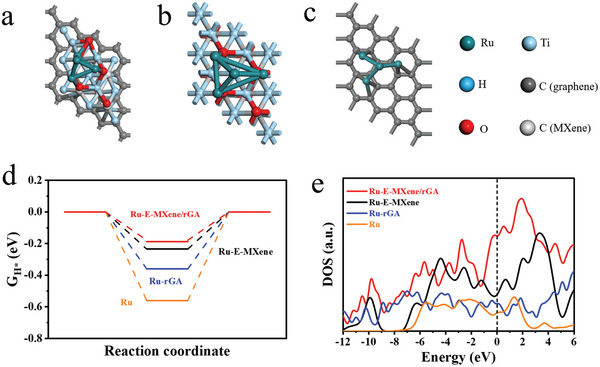
The simulative models of a) Ru‐E‐MXene/rGA, b) Ru‐E‐MXene, and c) Ru‐rGA. (gray, brown, red, and cyan balls represent Ti, C, O, Ru and H atoms). d) The calculated ΔG_H*_ values and e) TDOS of Ru‐E‐MXene/rGA, Ru‐E‐MXene, Ru‐rGA, and Ru.

**Figure** **6**a–c and Figure S18 (Supporting Information) show the calculation models of all catalysts, and the value of |ΔG_H*_| for Ru‐E‐MXene/rGA (0.18 eV) is smaller than that of Ru‐E‐MXene (0.24 eV), Ru‐rGA (0.36 eV) and Ru (0.56 eV) in Figure 6d, elucidating a better electrocatalytic HER activity. Too negative value of ΔG_H*_ for Ru NP and Ru‐rGA is caused by the strong adsorption and weak desorption of H*, which result in slow HER kinetics.^[^ ^35^ ^]^ Due to the electronic metal‐support interaction (EMSI) at the Ru/Ti_3_C_2_T_x_ interface, the binding energy of Ru‐H* is optimized.^[^ ^27^, ^36^ ^]^ In comparison with Ru‐E‐MXene, the |ΔG_H*_| value Ru‐E‐MXene/rGA is much closer to zero, attributing to the further optimization of H* adsorption energy by the interface interaction between MXene and graphene.^[^ ^12^ ^]^ Furthermore, the density of states (DOS) near the Fermi level for Ru‐E‐MXene/rGA and Ru‐E‐MXene is mainly contributed by the d orbitals of Ti and Ru, and Ru‐rGA is mainly from the d orbital of Ru (Figure S19, Supporting Information). The excellent catalytic HER activity‐enhanced active sites are assigned to the d‐electron dominance near the Fermi level.^[^ ^35^ ^]^ The total density of states (TDOS) of all the catalysts is shown in Figure 6e. The TDOS value of Ru‐E‐MXene/rGA near the Fermi level is much larger than that of Ru‐E‐MXene, Ru‐rGA, and Ru, indicating a higher carrier density and stronger ability to provide electrons. The enhanced conductivity of Ru‐E‐MXene/rGA can be attributed to a synergistic effect at the MXene/rGA interface to modulate electronic states near the Fermi level,^[^ ^37^ ^]^ which is beneficial to accelerate the hydrogen production process.

## 3 Conclusion

In summary, we successfully developed an efficient Ru‐E‐MXene/rGA catalyst with excellent HER performance under both acidic and alkaline conditions. Ru‐E‐MXene/rGA catalyst owns a large active surface area and abundant active sites. The formation of the MXene/rGA interface can strongly trap the positively charged Ru^3+^ ions and increase the electrical conductivity of the catalyst. In alkaline and acidic media, the overpotentials of Ru‐E‐MXene/rGA catalysts at 10 mA cm^−2^ are 42 and 62 mV, respectively, which is lower than other control samples. The small Tafel slope and *R*_ct_ values for Ru‐E‐MXene/rGA imply the fast kinetic behavior during the HER process. Ru‐E‐MXene/rGA exhibits excellent catalytic stability in both electrolytes and the boosted active sites are demonstrated by density functional theory calculation. This work provides an effective strategy to construct the hierarchically structured MXene‐based electrocatalyst for energy conversion applications.

## Conflict of Interest

The authors declare no conflict of interest.

## Supporting information

Supporting Information

## Data Availability

The data that support the findings of this study are available in the supplementary material of this article.
