# Supplementary material for: Interfacial Design of Ti3C2Tx MXene/Graphene Heterostructures Boosted Ru Nanoclusters with High Activity Toward Hydrogen Evolution Reaction
Source: Adv Sci (Weinh). 2024 Mar 29;11(22):2310013. doi: 10.1002/advs.202310013 (PMC11165527; doi:10.1002/advs.202310013)
Supplement: Supplementary file 1 — Supporting Information [file ADVS-11-2310013-s001.docx]

Supporting Information

**Interfacial design of hierarchical Ti_3_C_2_T_x_ MXene/graphene heterostructures boosted Ru nanocluster with high activity toward hydrogen evolution reaction**

*Xu Yu*, Yong Li, Chengang Pei,* *Yanhui Lu, Jung Kyu Kim, Ho Seok Park, and Huan Pang**

**Supplemental Experiments**

**S1.1 Chemicals**

Ruthenium chloride hydrate (RuCl_3_· *x*H_2_O), Ti_3_AlC_2_, lithium fluoride, HF solution (40%), KOH (95%), H_2_SO_4_, and Nafion solution (∼5 wt.% in a mixture of lower aliphatic alcohols and water) were purchased from Shanghai Aladdin Biochemical Technology Co., Ltd. GO were purchased from Nanjing Pioneer Nanomaterials Technology Co., Ltd., China. All chemicals used were used without further purification. All solutions were prepared with ultrapure water with a resistance of 18.2 MΩ (Thermo Fisher Scientific Co. LTD, USA).

**S1.2 Preparation of MXene and E-MXene (Ti_3_C_2_T_x_)**

The MXene was produced by the selective etching of Al from the MAX precursors. 1g Ti_3_AlC_2_ was slowly added to 10 mL of concentrated HF solution (40 wt.%), then stirred at 35 ℃ for 24 h. The mixture was washed with deionized water and ethanol several times and centrifuged at 3500 rpm until the pH of 7. Finally, the solution was centrifuged at 4000 rpm, and the precipitation was collected and freeze-dried for 3 days to obtain MXene powder. 500 mg of the MXene powder and 200 mg of LiF were added to 10 mL of deionized water and stirred for 3h to form a uniform suspension. The suspensions were treated with tip-sonication for 2.5 h under an ice bath. Then the exfoliated MXene suspension was washed by the centrifugation for few times and freeze-dried for 1 day to finally obtain the exfoliated MXene powder, named as E-MXene.

**S1.3 Preparation of Ru-E-MXene/rGA**

Typically, 40 mg of E-MXene powder, 10 mg of GO and 5 mg of ascorbic acid were added into 10 mL of ethylene glycol and applied with a magnetic-stirring for 10 min then followed by a ultrasonication for 30 min to obtain the Ti_3_C_2_T_x_ MXene/GA suspension. Then 20 mg RuCl_3_· *x*H_2_O was added into ethylene glycol, and the above two dispersions are mixed under magmatic stirring for 3 h. After that, the as-prepared mixture was transferred to a 50 mL of Teflon-lined stainless-steel autoclave and heated to 180 °C for 10 h. The black hydrogel was washed by DI water for several times and freeze-dried under vacuum condition for 3 days. The hierarchical structure was finally obtained and noted Ru-E-MXene/GA.

**S1.4 Preparation of Ru-E-MXene**

Typically, 50 mg of E-MXene powder and 5 mg of ascorbic acid were added into 10 mL of ethylene glycol and applied with a magnetic stirring for 10 min followed by an ultrasonication for 30 min to obtain the Ti_3_C_2_T_x_ MXene suspension. Then 20 mg RuCl_3_·*x*H_2_O  was added into ethylene glycol and the above two dispersions were mixed under magnetic stirring for 3 h. After that, the as-prepared mixture was transferred to a 50 mL Teflon-lined stainless-steel autoclave and heated to 180 °C for 10 h. The black precipitates were collected by washing with DI water and freeze-dried under vacuum conditions for 3 days.

**S1.5 Preparation of Ru-rGA**

50 mg of GO and 5 mg of ascorbic acid were dispersed in 10 mL of ethylene glycol and applied with a magnetic stirring for 10 min followed by an ultrasonication for 30 min to obtain the GA suspension. Then 20 mg RuCl_3_· *x*H_2_O was added into ethylene glycol, and the above two dispersions were mixed under magnetic stirring for 3 h. After that, the as-prepared mixture was transferred to a 50 mL Teflon-lined stainless-steel autoclave and heated to 180 °C for 10 h. The black hydrogel was washed with DI water several times and freeze-dried under vacuum conditions for 3 days. The hierarchical architecture was finally obtained and noted Ru-rGA.

**S1.6 Characterizations**

All samples were analyzed by scanning electron microscope (SEM, Hitachi, S-4800 II, Japan), transmission electron microscope (TEM), high-resolution transmission electron microscope (HRTEM, Philips, TECNAI 12, Holland), and the related element mapping analysis (200 kV Philips TECNAI G2 electron microscope). The KEVEX X-ray energy detector was used for energy color Scattered X-ray (EDS) analysis. Powder X-ray diffraction (XRD) patterns were recorded on a Bruker D8 Advance powder using a Cu Kα (λ=1.5405 Å) radiation source operating at 40 kV and 40 mA at a scanning rate of 5° min^-1^. Surface analysis of the sample was studied by X-ray photoelectron spectroscopy (XPS, Thermo Science, ESCALAB 250Xi, USA).

**S1.7 Electrochemical measurements**

The electrochemical measurements were performed by an electrochemical workstation (CHI660E) at room temperature. The HER performance was measured in a standard three-electrode configuration using the glass carbon electrode (GC, 3 nm diameter, 0.07 cm^2^) as a support working electrode. The graphite rod and the saturated calomel electrode (SCE) were utilized as the counter and reference electrodes, respectively. All potentials were referenced to the reversible hydrogen electrode (RHE) according to the following equation [E(RHE) = E(SCE) + 0.059 pH + 0.242 V]. The preparation of the catalyst ink was listed as follows: 5 mg of catalyst was firstly dispersed into the 950 µL ethanol a 50 µL Nafion solution (5wt%) under a 30 min bath-sonication. Then, 10 μL of the as-prepared catalyst ink was loaded onto a GC electrode and naturally dried at room temperature. The mass loading of the catalyst was about 0.7 mg cm^-2^. The catalyst was activated by cyclic voltammograms (CV) at 50 mV s^-1^ for 200 cycles. The polarization curve was measured by CV at 5 mV s^-1^ in both 1 M KOH and 0.5 M H_2_SO_4_. The double-layer capacitances (C_dl_) were determined by CV at scan rates from 5 to 50 mV s^-1^. Electrochemical impedance spectroscopy (EIS) was tested in a frequency range from 1000 kHz to 0.01 kHz by applying an AC voltage of 5 mV amplitude. Chronoamperometry (CA) was carried out at an overpotential of 10 mA cm^−2^ for 10 h to estimate the stability of the catalyst.

A gas-tight electrochemical cell with a gas burette was carried out to verify the faradaic yield of the catalyst by a typical three-electrode system. The working electrode was prepared by drop-casting catalyst suspension on a glassy carbon electrode with a surface area of 0.07 cm^-2^. The graphite rod and saturated calomel electrode (SCE) acted as the counter and reference electrodes, respectively. To reach the benchmark current density of 10 mA cm^-2^ at approximately potential (42 mV vs. RHE), the volume of the evolved gas was recorded synchronously. Thus, the faradaic yield was calculated from the ratio of the recorded gas volume to the theoretical gas volume during the charge passed through the electrode:

$$Faradaic yield=\frac{V_{\mathrm{experimental}}}{V_{\mathrm{theoretical}}}=\frac{V_{\mathrm{experimental}}}{\frac{1}{2}{\times\frac{Q}{F}\times V}_{m}}$$

Where Q is the charge passed through the electrode, F is Faraday constant (96485 C mol^-1^), the number 2 represents 2 mole electrons per mole H_2_, V_m_ is molar volume of gas (24.5 L mol^-1^, 298 K, 101 KPa).

**S1.8 Theoretical calculations**

The CASTEP module of the Materials Studio software (Accelrys Inc.) was employed for the quantum chemistry calculations^[^[^1^](#_ENREF_1)^]^. Perdew–Burke–Ernzerhof (PBE) approximation was selected as the generalized gradient approximation (GGA) method to calculate the exchange-correlation energy^[^[^2^](#_ENREF_2)^]^. The Broyden-Fletcher-GAldfarb-Shanno (BFGS) scheme was selected as the minimization algorithm. The vacuum space is adopted 15 Å above the surfaces to avoid periodic interactions. A cutoff energy of 450 eV was used for the plane-wave basis set, and a Monkhorst-Pack grid k-point of 2×2×1 was adopted to integrate the Brillouin zone^[^[^3^](#_ENREF_3)^]^. The iterative convergence of energy and force during optimization was set as 1×10^−5^ eV and 0.03 eV/Å, respectively. The self-consistence field (SCF) tolerance was set to 1.0×10^-5^ eV/atom. Accordingly, the Gibbs free energies of hydrogen adsorption (ΔG_H*_) were calculated as follows^[^[^4^](#_ENREF_4)^]^:

$$\Delta E_{H*}= E_{(slab+H*)}- E_{(slab)}-{1/2E}_{(H_{2})}$$

$$\Delta G_{H*}= \Delta E_{H*} + \Delta ZPE-T\Delta S$$

whereΔZPE stands for the change in zero-point energy and ΔS represents the entropy change. Here we take the value of 0.24 eV for ΔZPE - TΔS according to the previous report by Norskov et al^[^[^5^](#_ENREF_5)^]^.

**Supplementary Figures: Figure S1- S13**


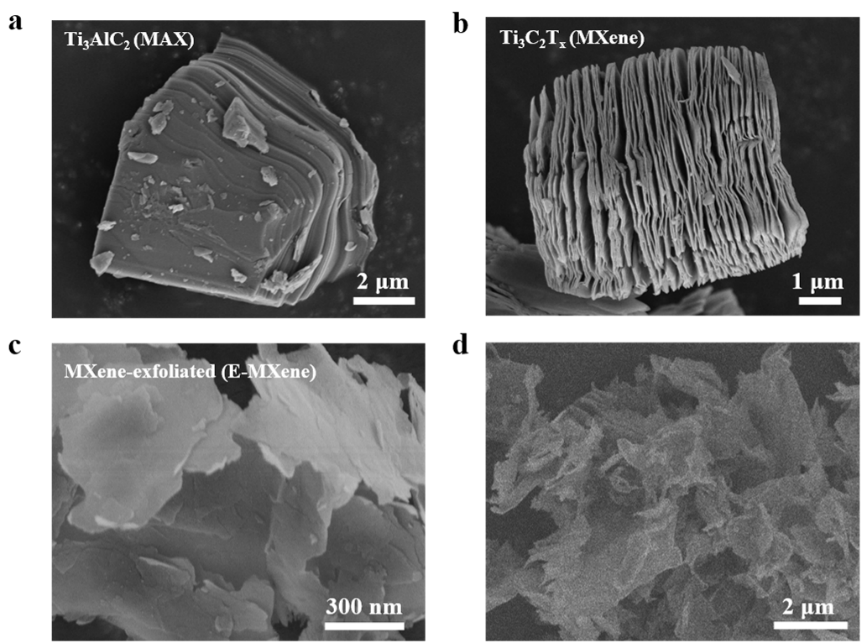


**Figure S1.** (a) SEM images of MAX. (b) SEM image of MXene. (c) SEM image of E-MXene. (d) SEM images of the Ru-E-MXene/rGA.


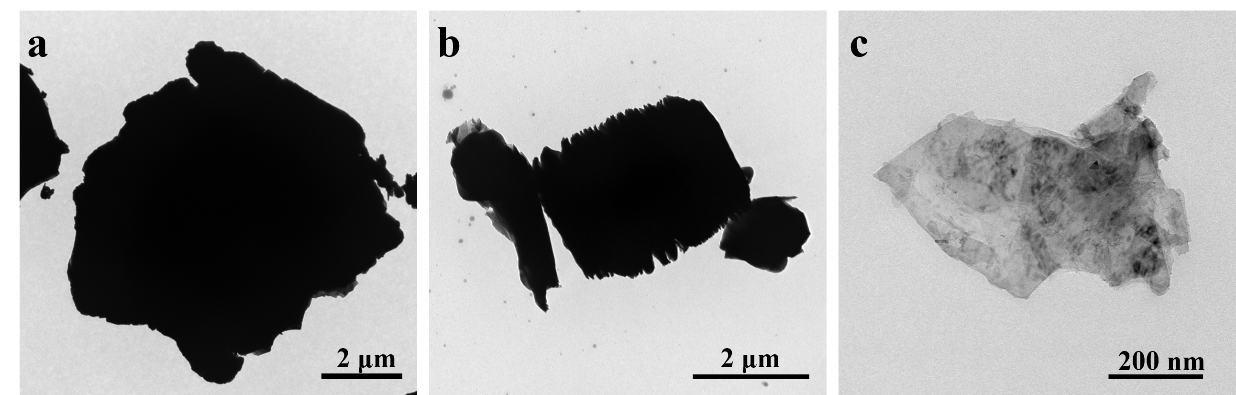


**Figure S2.** TEM of (a) MAX, (b) MXene-bulk, (c) E-MXene (Ti_3_C_2_Tx).


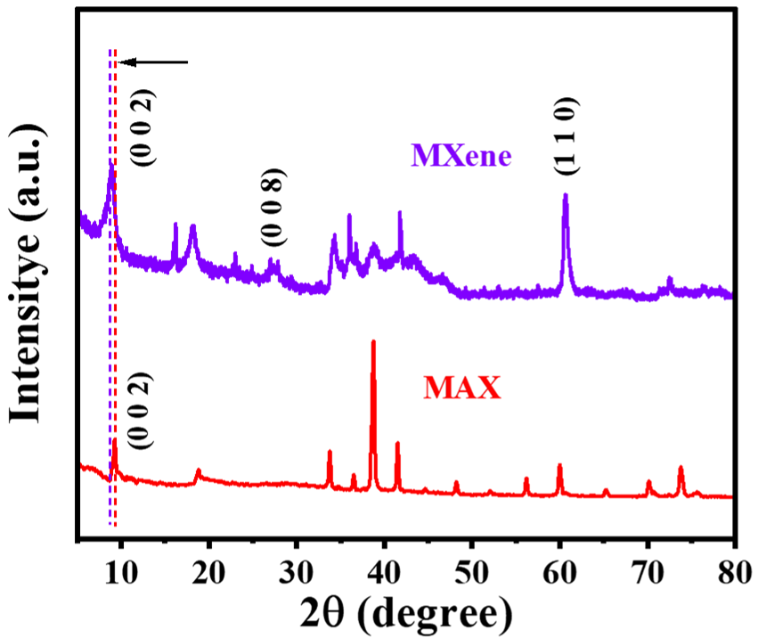


**Figure S3.** XRD patterns of MAX and MXene.


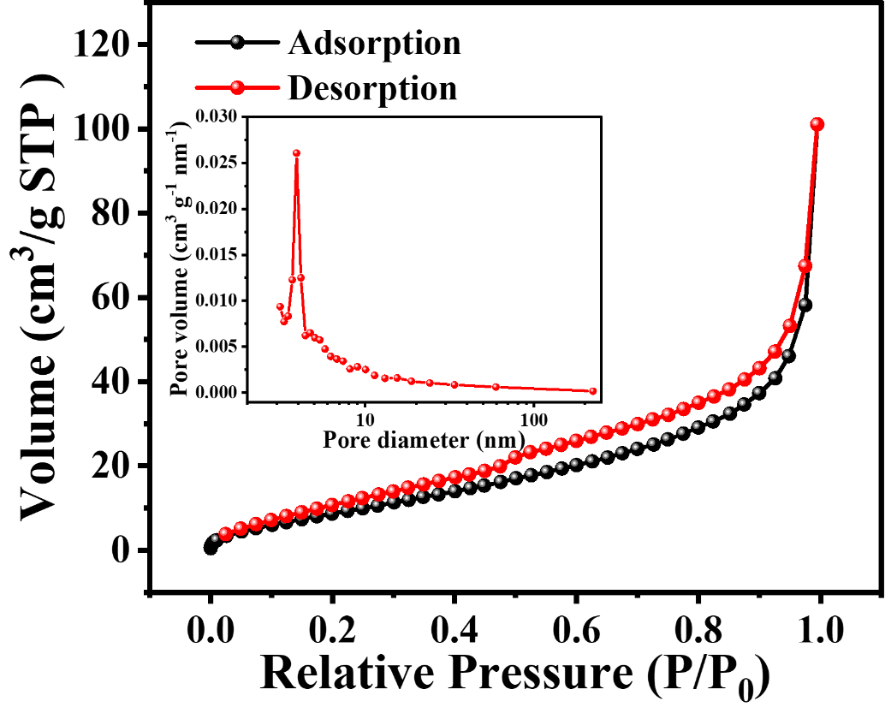


**Figure S4.** Nitrogen adsorption/desorption isotherm of Ru-E-MXene/rGA (inset: pore size distribution of Ru-E-MXene/rGA).


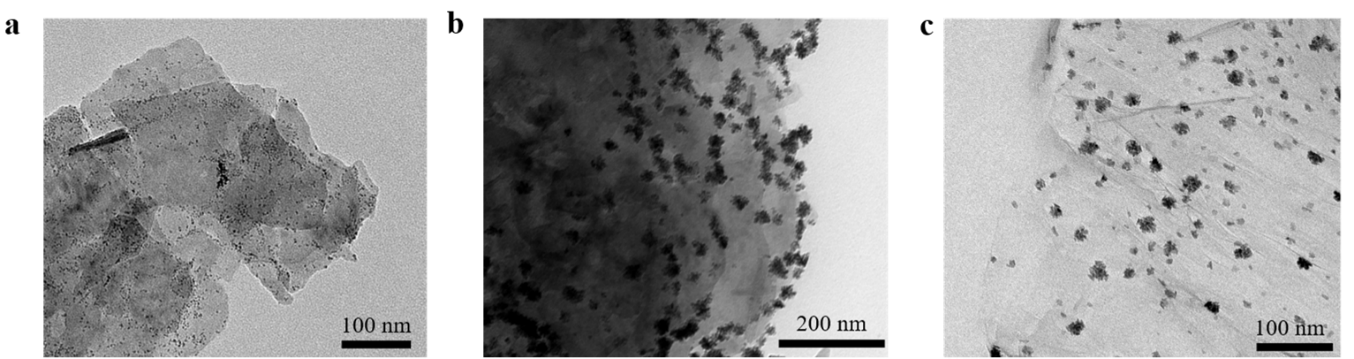


**Figure S5.** TEM of (a) Ru-E-MXene, (b) Ru-MXene, (c)Ru-rGA.


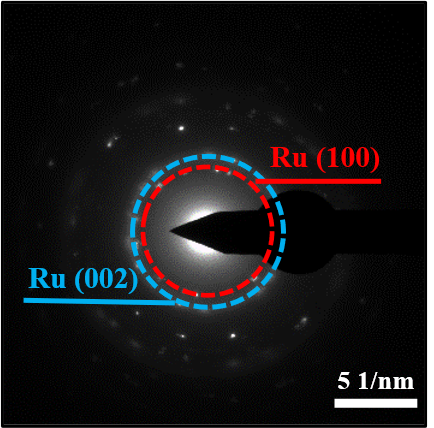


**Figure S6.** SAED pattern of Ru-E-MXene/rGA.


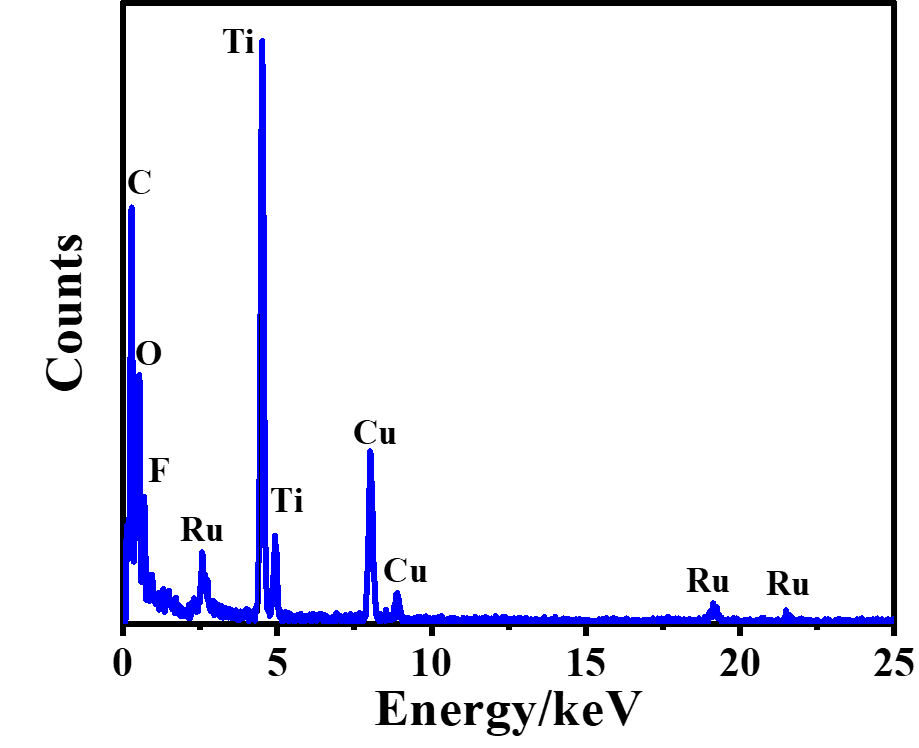


**Figure S7.** The EDS composition for Ru-E-MXene/rGA.


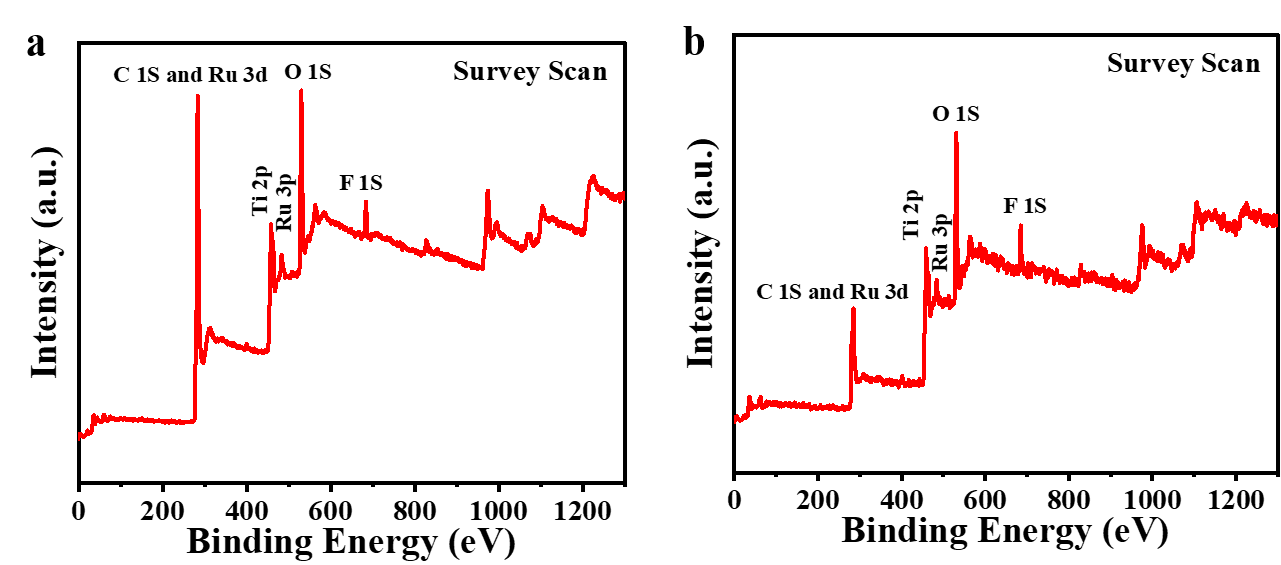


**Figure S8.** (a) The full scan XPS spectra of Ru-E-MXene/rGA. (b) The full scan XPS spectra of Ru-E-MXene.


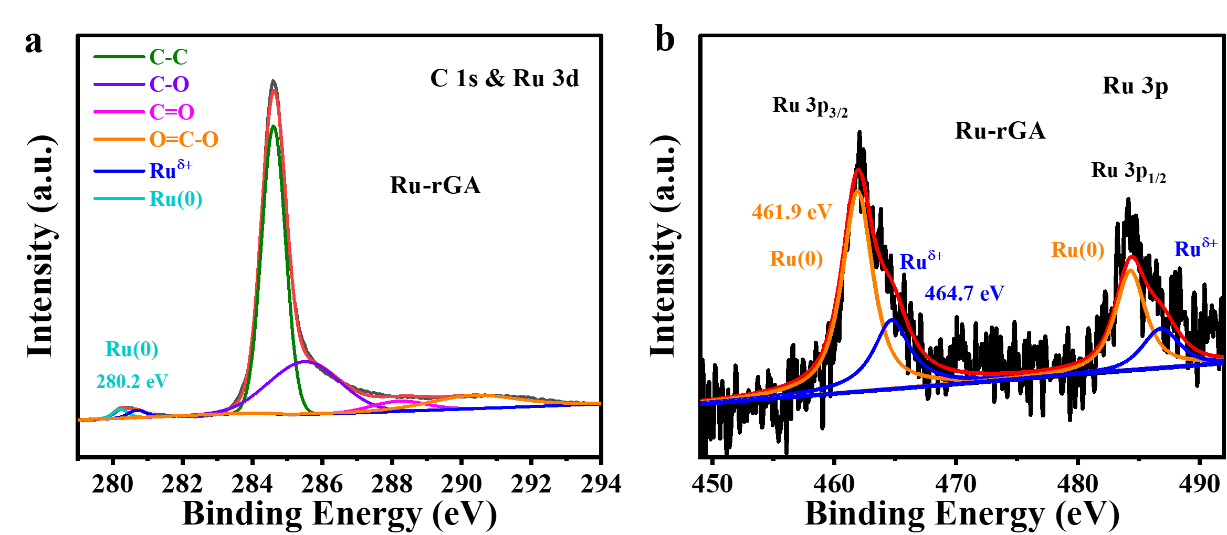


**Figure S9.** (a) C 1s & Ru 3d and (b) Ru 3p for Ru-rGA.


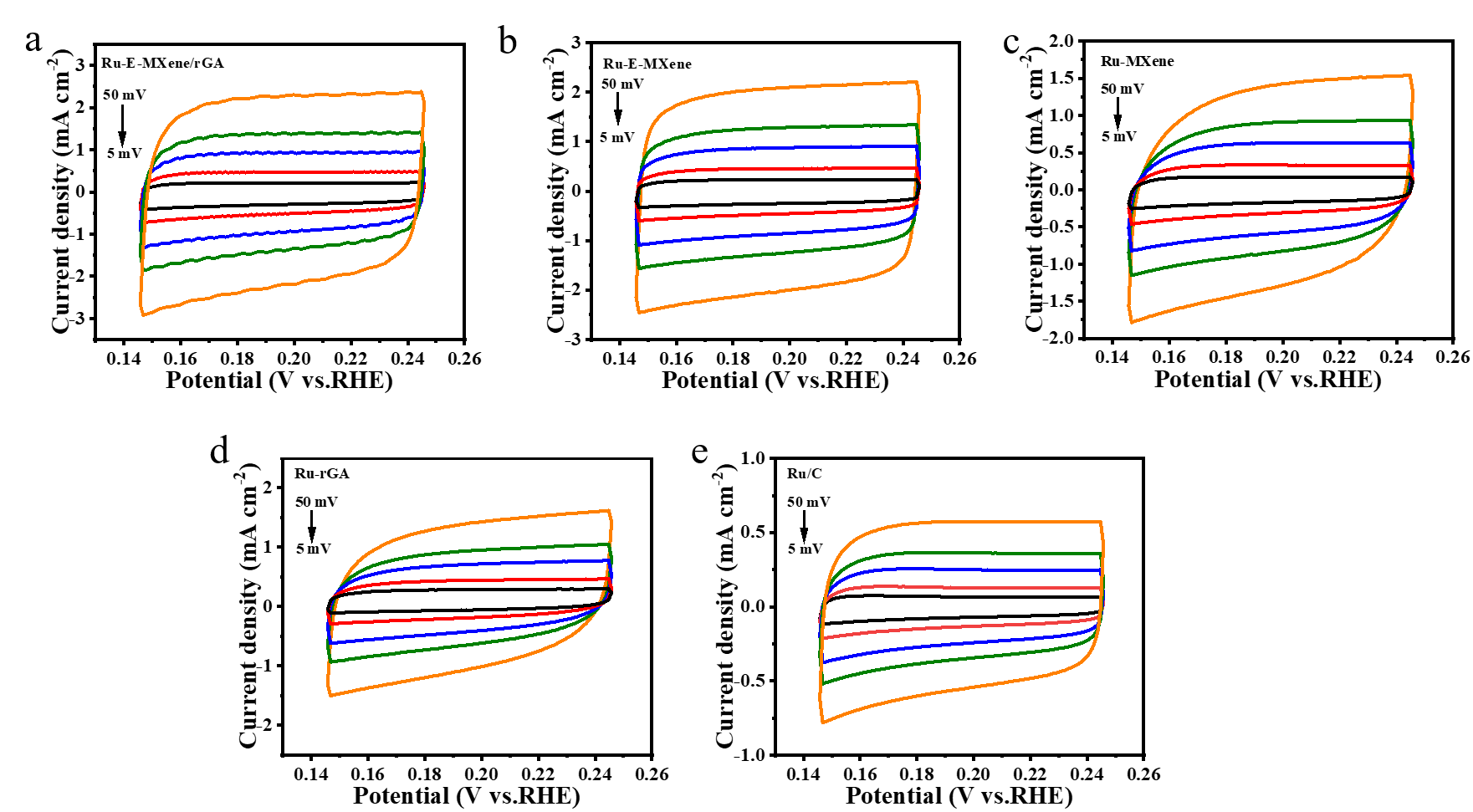


**Figure S10.** CV curves of (a) Ru-E-MXene/rGA, (b) Ru-E-MXene, (c) Ru-MXene, (d) Ru-rGA and (e) Ru/C at different scan rates in 1 M KOH for HER.


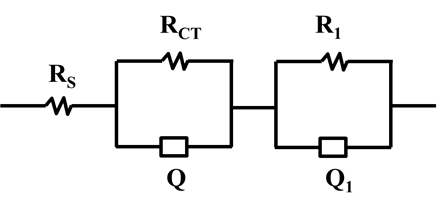


**Figure S11.** The equivalent circuit diagram. Where R_S_ is the solution resistance, R_ct_ is the charge transfer resistance, R_1_ is the interface resistance, and Q and Q1 are the equivalent capacitive element.


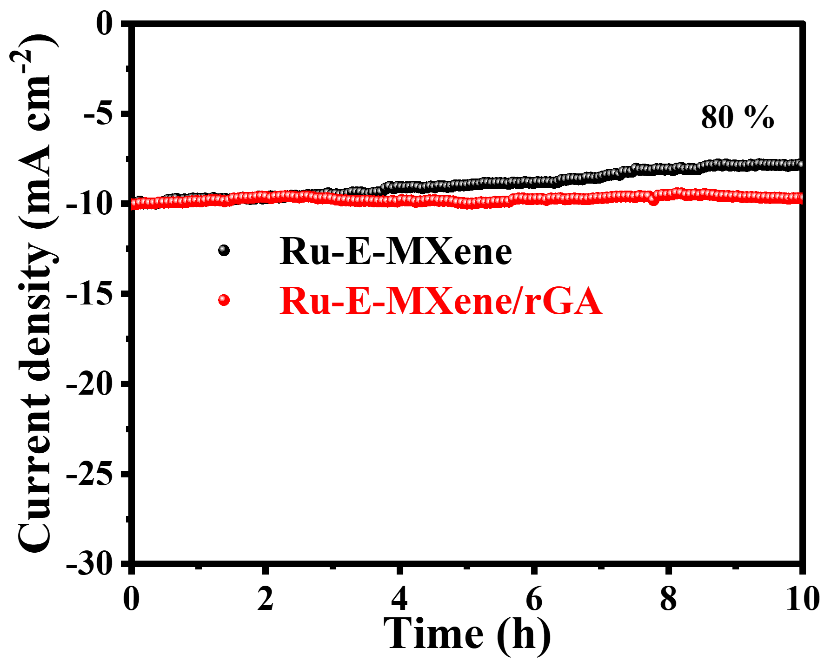


**Figure S12.** Stability of Ru-E-MXene and Ru-E-MXene/rGA electrocatalyst at 10 mA cm^−2^ for 10 h in alkaline solution.


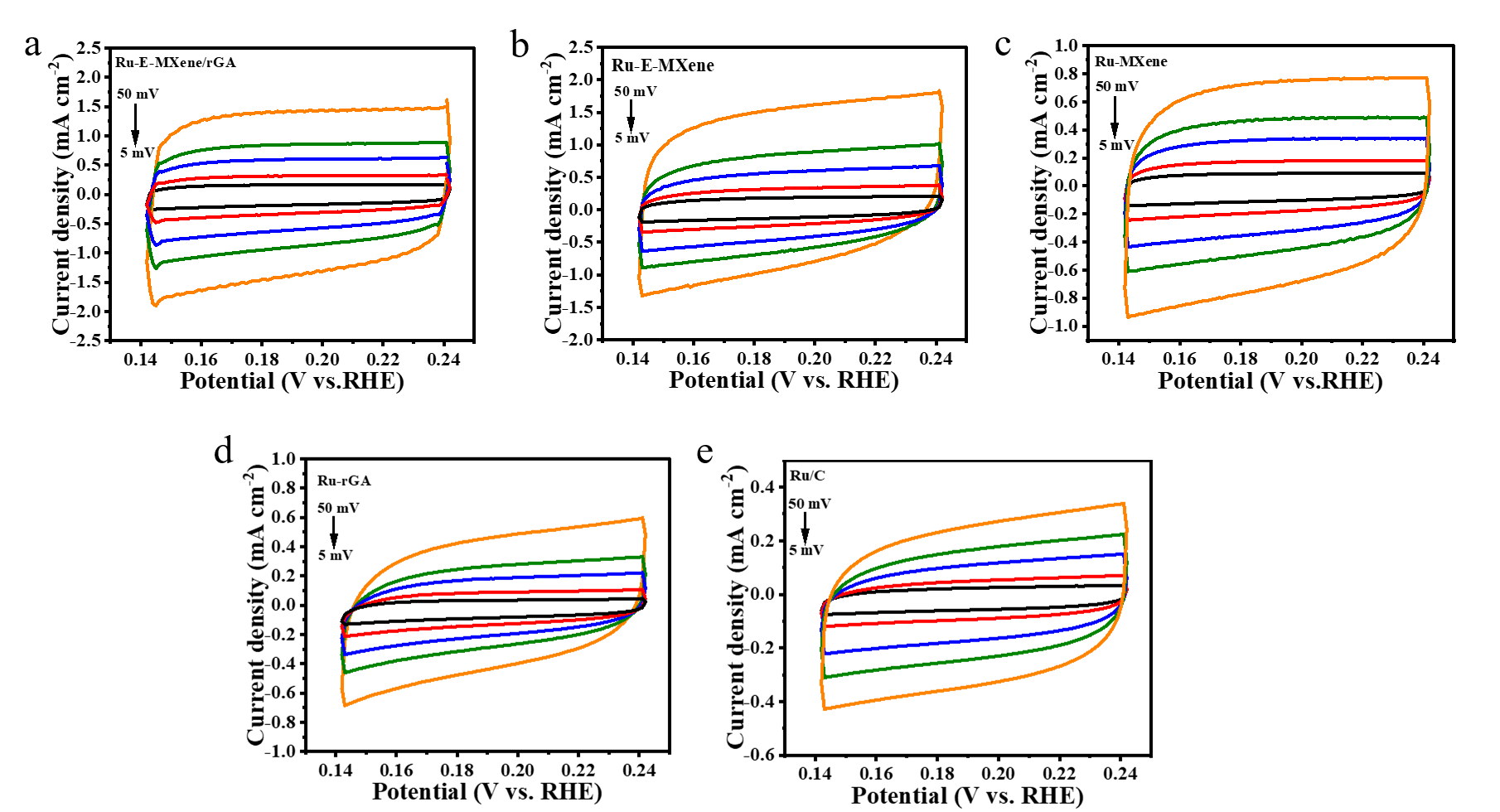


**Figure S13.** CV curves of (a) Ru-E-MXene/rGA, (b) Ru-E-MXene, (c) Ru-MXene, (d) Ru-rGA, and (e) Ru/C at different scan rates in 0.5 M H_2_SO_4_ for HER.


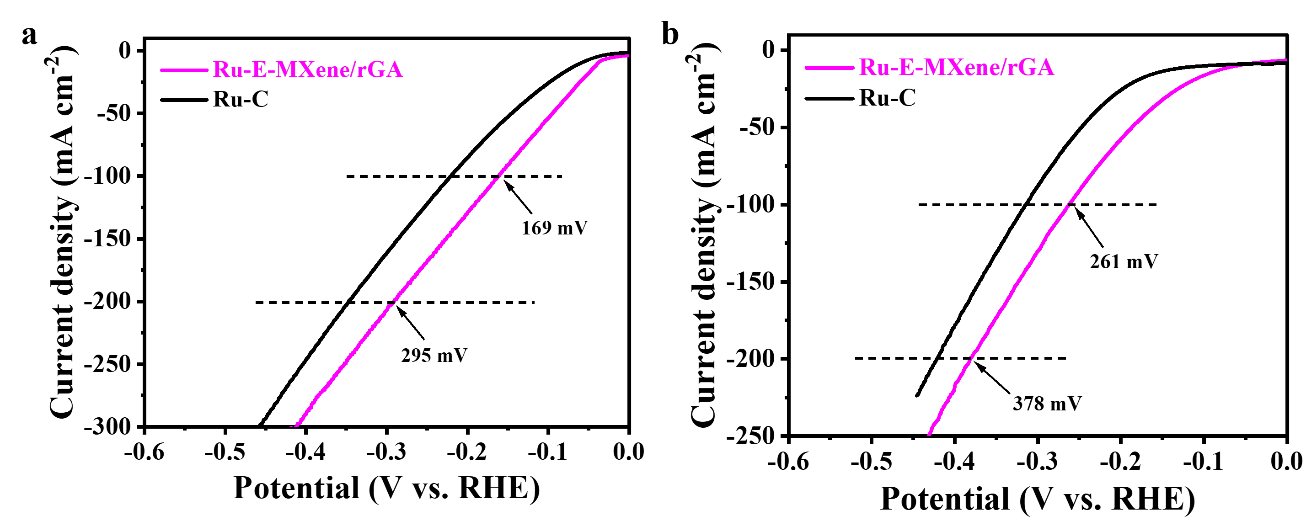


Figure S14. Polarization curves of Ru-E-MXene/rGA and Ru-C at high current density in alkaline solution (a) and acidic solution (b).

As shown in Figure S14, the overpotential of Ru-E-MXene/rGA is 169 and 295 mV at the high current density of 100 and 200 mA cm^-2^ in the alkaline electrolyte, which are much lower than these of Ru-C. Meanwhile, the overpotential of Ru-E-MXene/rGA is 261 and 378 mV at the high current density of 100 and 200 mA cm^-2^ in the acidic electrolyte, which are much lower than these of Ru-C.


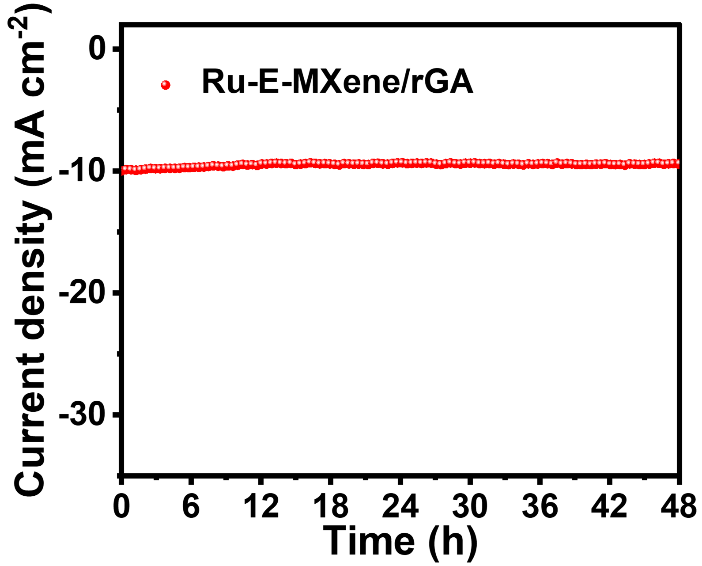


Figure S15. Stability of Ru-E-MXene/rGA electrocatalyst at 10 mA cm^−2^ for 48 h in alkaline solution.


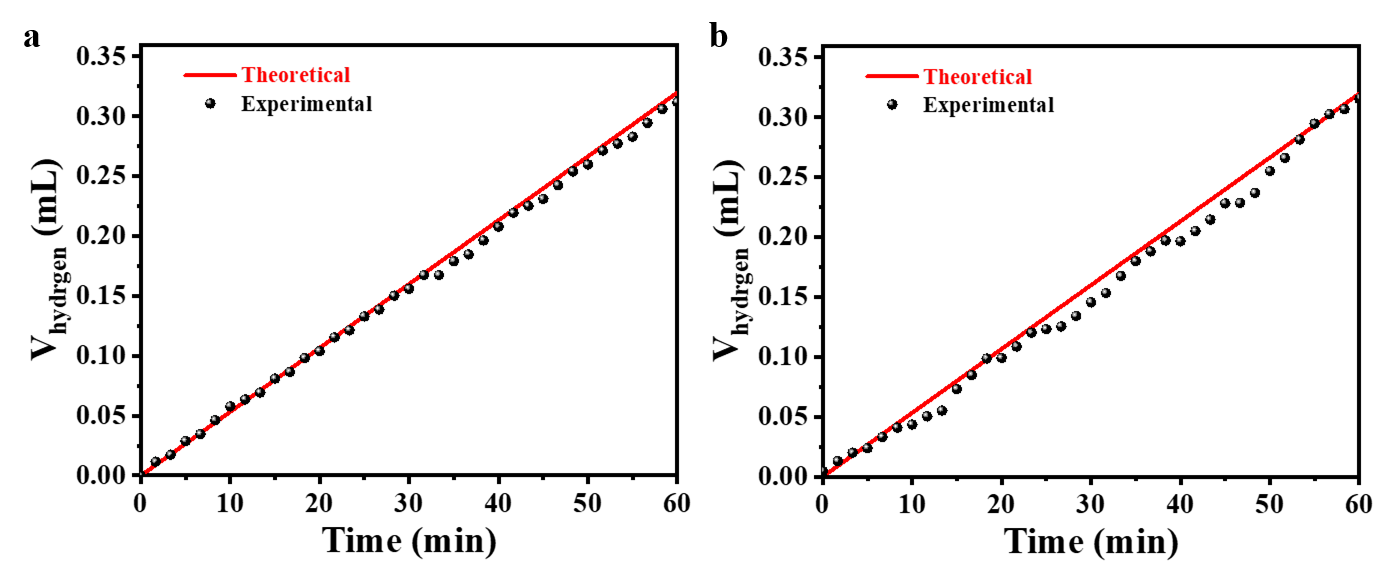


**Figure S16.** The amount of H_2_ theoretically calculated and experimentally measured versus time for Ru-E-MXene/rGA in (a)1 M KOH (at a constant overpotential of 42 mV) and (b) 0.5 M H_2_SO_4_ solution (at a constant overpotential of 62 mV), respectively. The theoretical line (red) represents the expected amounts of H_2_ assuming a nearly 100% Faradaic yield.


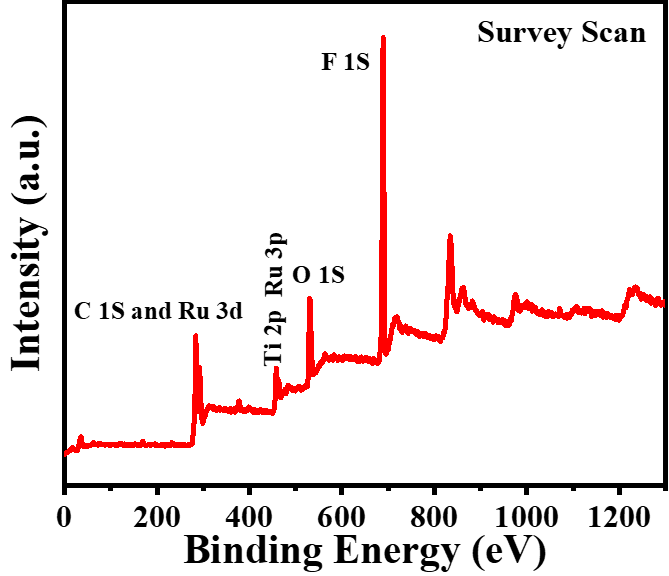


**Figure S17.** The full scan XPS spectra of Ru-E-MXene/rGA after stability test in 1M KOH.


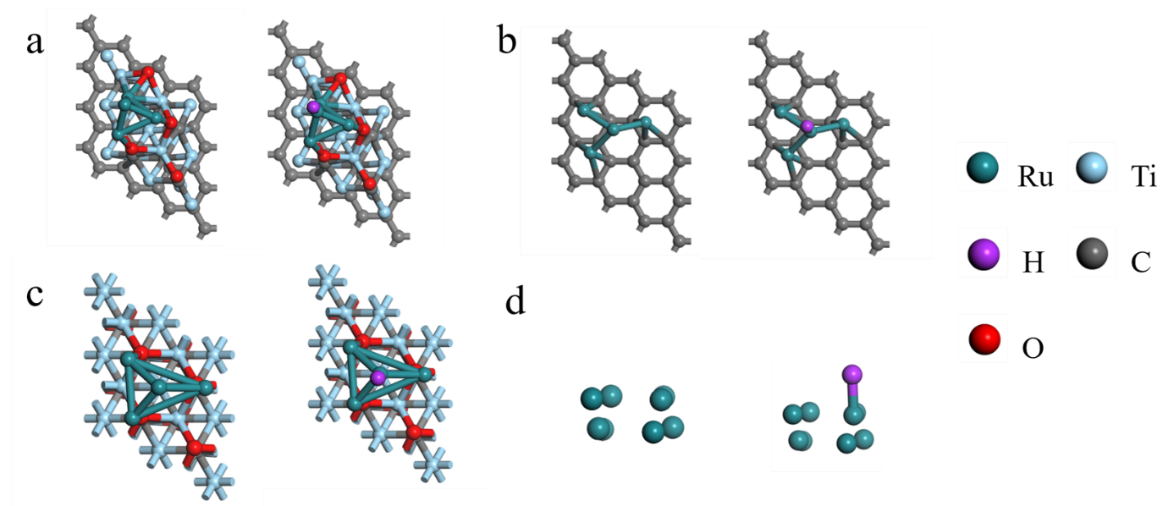


**Figure S18.** Theoretical structure models of H adsorbed on the Ru-E-MXene/rGA surface (a), Ru-GA surface (b), Ru-E-MXene surface (c), and Ru interface (d). (bule, gray, red, cyan and purple balls represent Ti, C, O, and H atoms).


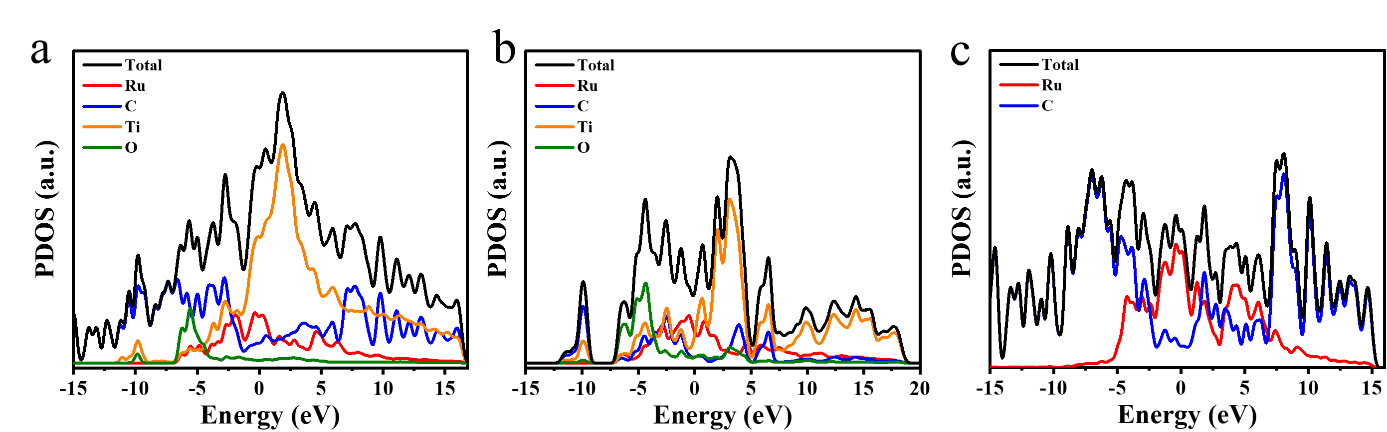


**Figure S19.** The projected densities of states (PDOS) of (a) Ru-E-MXene/rGA, (b) Ru-E-MXene, and (c) Ru-rGA.

**Supplementary Tables: Tables S1- S6**

**Table S1.** Specific surface area, pore volume, pore size of Ru-E-Mxene/rGA.

| Specific surface area (m^2^ g^-1^) | Pore Volume (cm^3^ g^-1^) | Pore Size (nm) |
| --- | --- | --- |
| 41.3 | 0.15 | 3.7 |

**Table S2.** The atomic ratio of all elements for Ru-E-Mxene/rGA.

| Elements | Ru | Ti | C | O | F |
| --- | --- | --- | --- | --- | --- |
| Atomic ratio % | 2.88 | 10.55 | 58.12 | 24.54 | 3.92 |

**Table S3.** The value of Cdl and ECSA for Ru-E-MXene/rGA and other control catalysts in 1 M KOH solution.

| Samples | *C_dl_*/mF cm^-2^ | *ECSA*/cm^2^ |
| --- | --- | --- |
| Ru-E-MXene/rGA | 44.1 | 77.2 |
| Ru-E-MXene | 40.4 | 70.7 |
| Ru-MXene | 26.4 | 46.2 |
| Ru-rGA | 23.3 | 40.8 |
| Ru-C | 10.9 | 19.1 |

**Table S4** The impedance fitting parameters of all catalysts for HER in 1 M KOH.

| Catalysts | R_s_/Ω cm^2^ | R_ct_/Ω cm^2^ | R_1_/ cm^2^ |
| --- | --- | --- | --- |
| Ru-E-MXene/rGA | 9.9 | 18.4 | 4.2 |
| Ru-E-MXene | 10.1 | 28.6 | 1.1 |
| Ru-MXene | 9.5 | 44.1 | 8.6 |
| Ru-rGA | 11.1 | 67 | 1.4 |
| Ru-C | 9.9 | 117.4 | 11.9 |

**Table S5.** Ru content in alkaline and acidic electrolytes after CA test for 10 h from ICP-OES.

| Solution | Concentration (ppm) | | |
| --- | --- | --- | --- |
|  | Ru | Ti | C |
| Alkaline (1 M KOH) | 0.002 | 0.005 | 1.184 |
| Acidic (0.5 M H_2_SO_4_) | 0.015 | 0.778 | 3.471 |

**Table S6** Comparison of electrocatalytic performance with other recently reported HER catalysts in 1M KOH solution.

| Catalyst | Overpotential (mV) | Electrolyte | Ref |
| --- | --- | --- | --- |
| Ru-E-MXene/rGA | 42 | 1M KOH | This work |
| RuSA-N-S-Ti_3_C_2_T_x_ | 99 | 1M KOH | ^[^[^6^](#_ENREF_6)^]^ |
| RuP_2_@NPC | 52 | 1M KOH | ^[^[^7^](#_ENREF_7)^]^ |
| CoRu-MoS_2_ | 52 | 1M KOH | ^[^[^8^](#_ENREF_8)^]^ |
| Ru@MoS_2_@CNTs | 50 | 1M KOH | ^[^[^9^](#_ENREF_9)^]^ |
| Ru/C_3_N_4_/C | 79 | 1M KOH | ^[^[^10^](#_ENREF_10)^]^ |
| Pt@PCM | 139 | 1M KOH | ^[^[^11^](#_ENREF_11)^]^ |
| Ru@NG-750 | 40 | 1M KOH | ^[^[^12^](#_ENREF_12)^]^ |
| Ru@WNO-C | 24 | 1M KOH | ^[^[^13^](#_ENREF_13)^]^ |
| Ru SAs-NiP | 57 | 1M KOH | ^[^[^14^](#_ENREF_14)^]^ |
| Ru-Ru_2_P-NPC | 46 | 1M KOH | ^[^[^15^](#_ENREF_15)^]^ |
| SA-Ru-MoS_2_ | 51 | 1M KOH | ^[^[^16^](#_ENREF_16)^]^ |
| Ru-NGC | 65 | 1M KOH | ^[^[^17^](#_ENREF_17)^]^ |

**Table S7.** The values of C_dl_ and ECSA for Ru-E-MXene/rGA and other control catalysts in 0.5 M H_2_SO_4_ solution.

| Samples | *C_dl_*/mF cm^-2^ | *ECSA*/cm^2^ |
| --- | --- | --- |
| Ru-E-MXene/rGA | 26.8 | 46.9 |
| Ru-E-MXene | 23.6 | 41.3 |
| Ru-MXene | 14 | 24.5 |
| Ru-rGA | 8.5 | 14.9 |
| Ru-C | 5.8 | 10.2 |

**Table S8.** The impedance fitting parameters of all catalysts for HER in 0.5 M H_2_SO_4_ solution.

| Catalysts | R_s_/Ω cm^2^ | R_ct_/Ω cm^2^ | R_1_/Ω cm^2^ |
| --- | --- | --- | --- |
| Ru-E-MXene/rGA | 9.4 | 29.2 | 3.4 |
| Ru-E-MXene | 9.7 | 33.9 | 1.9 |
| Ru-MXene | 10.2 | 57.2 | 4.3 |
| Ru-rGA | 9.0 | 65.1 | 0.8 |
| Ru-C | 9.2 | 132.1 | 20.4 |

**Table S9** Comparison of electrocatalytic performance with other recently reported HER catalysts in 0.5 M H_2_SO_4_ solution.

| Catalyst | Overpotential (mV) | Electrolyte | Ref |
| --- | --- | --- | --- |
| Ru-E-MXene/rGA | 62 | 0.5 M H_2_SO_4_ | This work |
| RuSA-N-S-Ti_3_C_2_T_x_ | 76 | 0.5 M H_2_SO_4_ | ^[^[^6^](#_ENREF_6)^]^ |
| Ru/C_3_N_4_/C | 75 | 0.5 M H_2_SO_4_ | ^[^[^10^](#_ENREF_10)^]^ |
| Pt@PCM | 105 | 0.5 M H_2_SO_4_ | ^[^[^11^](#_ENREF_11)^]^ |
| Ru@NG-750 | 90 | 0.5 M H_2_SO_4_ | ^[^[^12^](#_ENREF_12)^]^ |
| Ru@WNO-C | 172 | 0.5 M H_2_SO_4_ | ^[^[^13^](#_ENREF_13)^]^ |
| Ru SAs-NiP | 125 | 0.5 M H_2_SO_4_ | ^[^[^14^](#_ENREF_14)^]^ |
| Ru/d-NPC | 68 | 0.5 M H_2_SO_4_ | ^[^[^18^](#_ENREF_18)^]^ |
| Ru@B-Ti_3_C_2_T_x_ | 62.9 | 0.5 M H_2_SO_4_ | ^[^[^19^](#_ENREF_19)^]^ |
| Mxene@Pt/SWCT | 62 | 0.5 M H_2_SO_4_ | ^[^[^20^](#_ENREF_20)^]^ |
| CoRu/CNTs@Ti_3_C_2_ | 74 | 0.5 M H_2_SO_4_ | ^[^[^21^](#_ENREF_21)^]^ |
| RuNi/CQDs | 58 | 0.5 M H_2_SO_4_ | ^[^[^22^](#_ENREF_22)^]^ |
| Ru-SA/Ti_3_C_2_Tx | 70 | 0.5 M H_2_SO_4_ | ^[^[^23^](#_ENREF_23)^]^ |

**References**

[1] S. J. Clark, M. D. Segall, C. J. Pickard, P. J. Hasnip, M. J. Probert, K. Refson, M. C. Payne, *Z Kristallogr Cryst Mater* **2005**, *220*, 567-570.

[2] J. P. Perdew, K. Burke, M. Ernzerhof, *Phys. Rev. Lett.* **1996**, *77*, 3865-3868.

[3] G. Li, T. Sun, H.-J. Niu, Y. Yan, T. Liu, S. Jiang, Q. Yang, W. Zhou, L. Guo, *Adv. Funct. Mater.* **2023**, *33*, 2212514.

[4] X. Luo, P. X. Ji, P. Y. Wang, X. Tan, L. Chen, S. C. Mu, *Adv. Sci.* **2022**, *9*, 2104846.

[5] J. K. Norskov, T. Bligaard, A. Logadottir, J. R. Kitchin, J. G. Chen, S. Pandelov, J. K. Norskov, *J. Electrochem. Soc.* **2005**, *152*, J23-J26.

[6] V. Ramalingam, P. Varadhan, H. C. Fu, H. Kim, D. L. Zhang, S. M. Chen, L. Song, D. Ma, Y. Wang, H. N. Alshareef, J. H. He, *Adv. Mater.* **2019**, *31*, 1903841.

[7] Z. H. Pu, I. S. Amiinu, Z. K. Kou, W. Q. Li, S. C. Mu, *Angew. Chem., Int. Ed.* **2017**, *56*, 11559-11564.

[8] I. S. Kwon, T. T. Debela, I. H. Kwak, Y. C. Park, J. Seo, J. Y. Shim, S. J. Yoo, J. G. Kim, J. Park, H. S. Kang, *Small* **2020**, *16*, 2000081.

[9] X. Zhang, F. Zhou, S. Zhang, Y. Y. Liang, R. H. Wang, *Adv. Sci.* **2019**, *6*, 1900090.

[10] Y. Zheng, Y. Jiao, Y. H. Zhu, L. H. Li, Y. Han, Y. Chen, M. Jaroniec, S. Z. Qiao, *J. Am. Chem. Soc.* **2016**, *138*, 16174-16181.

[11] H. B. Zhang, P. F. An, W. Zhou, B. Y. Guan, P. Zhang, J. C. Dong, X. W. Lou, *Sci. Adv.* **2018**, *4*, aao6657.

[12] L. Bai, Z. Y. Duan, X. D. Wen, R. Si, Q. Q. Zhang, J. Q. Guan, *ACS Catal.* **2019**, *9*, 9897-9904.

[13] G. Meng, H. Tian, L. X. Peng, Z. H. Ma, Y. F. Chen, C. Chen, Z. W. Chang, X. Z. Cui, J. L. Shi, *Nano Energy* **2021**, *80*, 105531.

[14] K. L. Wu, K. A. Sun, S. J. Liu, W. C. Cheong, Z. Chen, C. Zhang, Y. Pan, Y. S. Cheng, Z. W. Zhuang, X. W. Wei, Y. Wang, L. R. Zheng, Q. H. Zhang, D. S. Wang, Q. Peng, C. Chen, Y. D. Li, *Nano Energy* **2021**, *80*, 105467.

[15] J. Y. Yu, G. X. Li, H. Liu, L. L. Zhao, A. Z. Wang, Z. Liu, H. D. Li, H. Liu, Y. Y. Hu, W. J. Zhou, *Adv. Funct. Mater.* **2019**, *29*, 1901154.

[16] J. Wang, W. H. Fang, Y. Hu, Y. H. Zhang, J. Q. Dang, Y. Wu, B. Z. Chen, H. Zhao, Z. X. Li, *Appl. Catal. B-Environ.* **2021**, *298*, 120490.

[17] Q. Song, X. Z. Qiao, L. Z. Liu, Z. J. Xue, C. H. Huang, T. Wang, *Chem. Commun.* **2019**, *55*, 965-968.

[18] W. Q. Li, H. Zhang, K. Zhang, W. X. Hu, Z. Z. Cheng, H. P. Chen, X. Feng, T. Peng, Z. K. Kou, *Appl. Catal. B-Environ.* **2022**, *306*, 121095.

[19] M. Bat-Erdene, M. Batmunkh, B. Sainbileg, M. Hayashi, A. S. R. Bati, J. D. Qin, H. J. Zhao, Y. L. Zhong, J. G. Shapter, *Small* **2021**, *17*, 2102218.

[20] C. Cui, R. F. Cheng, H. Zhang, C. Zhang, Y. H. Ma, C. Shi, B. B. Fan, H. L. Wang, X. H. Wang, *Adv. Funct. Mater.* **2020**, *30*, 2000693.

[21] Z. Li, M. Liu, S. Ma, Z. Jia, D. Jia, *ACS Appl. Energ. Mater.* **2023**, *6*, 1519-1525.

[22] Y. Liu, X. Li, Q. H. Zhang, W. D. Li, Y. Xie, H. Y. Liu, L. Shang, Z. Y. Liu, Z. M. Chen, L. Gu, Z. Y. Tang, T. R. Zhang, S. Y. Lu, *Angew. Chem., Int. Ed.* **2020**, *59*, 1718-1726.

[23] H. G. Liu, Z. Hu, Q. L. Liu, P. Sun, Y. F. Wang, S. L. Chou, Z. Z. Hu, Z. Q. Zhang, *J. Mater. Chem. A* **2020**, *8*, 24710-24717.
